# Tunable Narrow-Linewidth Si_3_N_4_ External-Cavity Semiconductor Laser Based on an Asymmetric Bezier Triple-Ring Resonator

**DOI:** 10.3390/s26134070

**Published:** 2026-06-26

**Authors:** Tong Wang, Yuchen Hu, Wen Zhou, Ye Wang, Xiangjun Xin

**Affiliations:** 1 State Key Laboratory of Integrated Chips and Systems, Fudan University, Shanghai 200433, China; 24210720274@m.fudan.edu.cn (T.W.); huyuchen@fudan.edu.cn (Y.H.); 2 School of Future Information Science and Technology, Fudan University, Shanghai 200433, China; 3 China Mobile Jiangsu Company Limited, Nanjing 210029, China; wangyesgs@js.chinamobile.com; 4 IEIT Systems (Beijing) Co., Ltd., Beijing 100095, China; xinxiangjun@ieisystem.com

**Keywords:** external cavity, asymmetric Bezier bend waveguide, ring, narrow linewidth

## Abstract

This paper presents an AI-assisted design of a tunable narrow-linewidth external cavity laser based on an asymmetric Bezier cascaded triple-ring resonator. To address the high bending loss, limited quality factor, and footprint–loss trade-off in conventional external cavity ring resonators, asymmetric Bezier waveguide bends are introduced and optimized using particle swarm optimization, enabling smoother mode evolution and reducing bend loss. On this basis, this paper constructs a triple-ring-coupled external cavity structure to further enhance the mode-selection capability and filtering performance of the resonant cavity. Simulation results indicate that the asymmetric Bezier resonator optimized using particle swarm optimization can effectively reduce cavity loss and improve the resonator quality factor while maintaining a compact device footprint. Theoretical analysis indicates that the designed laser based on the proposed external cavity can achieve a linewidth of approximately 390 Hz. This work provides a feasible approach for designing high-performance, low-loss, and narrow-linewidth-integrated lasers, and is of significance for the development of integrated photonic laser sources.

## 1. Introduction

Semiconductor lasers with narrow linewidth and wide tunability are of great importance for coherent optical communications [1,2], LiDAR [3,4], high-resolution spectroscopy [5,6], precision metrology [7,8], and integrated sensing [9,10]. With the rapid development of photonic integration, achieving on-chip laser sources that simultaneously combine narrow linewidth, wide tunability, a compact footprint, and high integration density has become a central challenge in integrated optoelectronics. Among the various approaches, external cavity semiconductor lasers (ECLs) are widely regarded as an effective solution. Their performance is largely determined by the external cavity filtering and feedback structure. Microring resonators (MRRs) are particularly attractive for this purpose because of their compact footprint, high quality factor, and inherent wavelength selectivity. Moreover, their large free spectral range, together with the optical Vernier effect, enables broad wavelength tuning, making MRR-based ECLs a promising platform for integrated narrow-linewidth, widely tunable lasers. Initial chip-scale external cavity lasers were predominantly developed on monolithic III–V platforms, in which both the optical gain section and the passive guiding structures were integrated within a single III–V epitaxial structure. These devices have achieved on-chip tuning spans of approximately 60 nm and intrinsic linewidths on the order of several tens of kilohertz [11]. Nevertheless, the comparatively large propagation loss in the passive III–V waveguides severely limits further linewidth narrowing. The emergence of heterogeneous III–V/Si and III–V/Si_3_N_4_ integration has opened a new pathway for improving laser performance by combining efficient III–V gain media with low-loss passive photonic circuits. In particular, Si_3_N_4_ stands out as a compelling platform owing to its extremely low optical loss, broad transparency range, and compatibility with CMOS fabrication, all of which are advantageous for implementing low-loss external cavities and high-Q resonant feedback elements.

Recent advances in hybrid Si_3_N_4_-based ECLs have highlighted the strong potential of this platform for realizing tunable narrow-linewidth lasers. In 2023, Chen et al. [12] reported a hybrid-integrated Si_3_N_4_ external cavity laser employing a gain chip and a dual-ring narrowband filter. The device enabled full C-band operation with a 55 nm tuning range and a side-mode suppression ratio above 50 dB, while delivering as much as 220 mW output power and a linewidth below 8 kHz. While this work demonstrated the promise of the approach, further improvements at the system level may still be required for deployment in demanding long-haul coherent transmission systems. In 2024, Wu et al. [13] employed defect-assisted high-Q MRRs and optimized the external cavity feedback, reducing the intrinsic linewidth to below 10 Hz over a tuning range from 1525 to 1565 nm. This result represented a major step forward in coherence performance, but the accessible tuning range remained relatively narrow. A hybrid ECL was reported by Fan et al. [14] in 2025, in which wavelength selection was realized using a dual-ring Vernier filter combined with a tunable Sagnac loop reflector. The laser achieved 90 nm of tunability with an intrinsic linewidth of 23.8 kHz, but this linewidth still falls short of the requirements for space coherent optical communication systems. Taken together, these studies indicate that, despite substantial progress, it remains difficult for integrated Si_3_N_4_-based ECLs to simultaneously achieve wide tunability, ultranarrow linewidth, and high output power.

Among the factors limiting further performance improvement, the geometry of the feedback resonator plays a particularly important role. Extending the effective optical length of the external cavity is beneficial for linewidth narrowing, but it may also introduce additional propagation loss [15]. Likewise, strong wavelength selectivity usually relies on high-Q resonators, whose performance is strongly affected by waveguide loss and structural compactness [16,17]. In compact MRR or racetrack resonators, bending loss and mode mismatch between straight and curved waveguide sections can significantly degrade the resonator Q factor and increase cavity loss [18,19]. Therefore, optimizing the bend profile has become an effective strategy for improving compact feedback resonators. Previous studies have shown that bend engineering can effectively reduce loss and improve resonator performance. For example, on the SOI platform, Euler-gradient resonant rings have been employed to improve the performance of narrow-linewidth ECLs [20].

Compared with conventional bend profiles, including circular and Euler-type designs, asymmetric Bezier bend waveguides provide greater geometric freedom by independently optimizing the two boundaries of the curved waveguide. This design can effectively reduce bending loss and mode mismatch without significantly increasing the device footprint, making it suitable for the realization of low-loss, high-Q resonators in integrated ECLs. In addition, intelligent optimization methods, particularly particle swarm optimization (PSO), can be introduced to optimize the Bezier control points and structural parameters for improved optical performance.

In this work, we propose an AI-assisted tunable narrow-linewidth Si_3_N_4_ ECL based on an asymmetric Bezier cascaded triple-ring resonator. By combining PSO-optimized low-loss asymmetric Bezier racetrack resonators with a highly selective triple-ring feedback structure, the proposed design simultaneously improves the resonator quality factor, suppresses side modes, and enhances the effective optical length of the external cavity. Simulation results show that the laser achieves a tuning range of 100 nm, a side-mode suppression ratio (SMSR) exceeding 80 dB, and an output power of 34 mW. The Lorentzian linewidth is 390 Hz at 1547 nm and remains within the sub-kHz range across the full tuning range. Overall, the proposed AI-assisted design provides a balanced combination of wide tunability, narrow linewidth, high SMSR, and relatively high output power and therefore offers a promising solution for high-performance integrated laser sources.

## 2. Theoretical Analysis

### 2.1. Tuning Range and SMSR

A single resonant ring is generally unable to provide a sufficiently wide tuning range because its free spectral range is limited by the cavity length. To overcome this limitation, the Vernier effect [21,22] can be employed by cascading resonant rings with slightly different free spectral ranges. In such a configuration, only the wavelengths simultaneously satisfying the resonance conditions of all rings can form the dominant transmission peaks, leading to a much larger effective free spectral range, denoted as $FSR_{eff}$. For a two-ring Vernier filter, $FSR_{eff}$ can be expressed as:

(1) $$FSR_{eff}=\frac{FSR_{1}\cdot FSR_{2}}{\left|FSR_{1}-FSR_{2}\right|}$$

where $FSR_{1}$ and $FSR_{2}$ are the free spectral ranges of the two resonant rings, respectively. The free spectral range of an individual resonant ring can be written as:

(2) $$FSR_{m}=\frac{\lambda^{2}}{n_{g}L_{m}}$$

where λ is the central wavelength, $n_{g}$ is the group refractive index of the waveguide, and $L_{m}$ is the perimeter of the m-th resonant ring.

**Figure 1:**
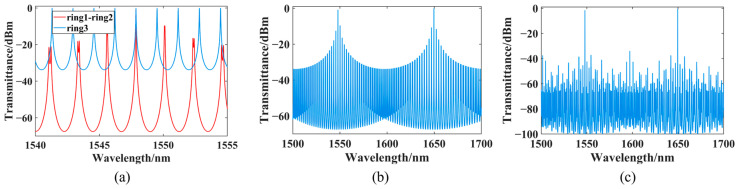
(**a**) Spectral responses of the dual-ring Vernier filter and the third ring; (**b**) the dual-ring filter provides a tuning range of 100 nm, while its SMSR is only 10 dB; (**c**) after adding the third ring, the SMSR is increased to 37 dB while maintaining the 100 nm tuning range.

Although a dual-ring Vernier structure can effectively enlarge the tuning range, the SMSR may degrade when the spacing between adjacent composite peaks becomes too small. To address this issue, a third resonant ring is introduced on the basis of the dual-ring Vernier structure to further suppress side modes. As illustrated in Figure 1a, the red curve represents the composite transmission spectrum generated by the first two rings, while the blue curve corresponds to the transmission response of the third ring. By properly choosing the perimeter of the third ring, its transmission minimum can overlap with the lateral peak of the dual-ring composite spectrum, thereby reducing the intensity of the side mode. Figure 1b shows the composite transmission spectrum of the dual-ring Vernier structure over a wide wavelength range. After introducing the third ring, the side modes are effectively suppressed, while the main resonance peaks are retained, as shown in Figure 1c. Therefore, the external cavity can simultaneously achieve an effective FSR of 100 nm and an SMSR of 37 dB.

### 2.2. Theoretical Analysis of Laser Linewidth

**Figure 2:**
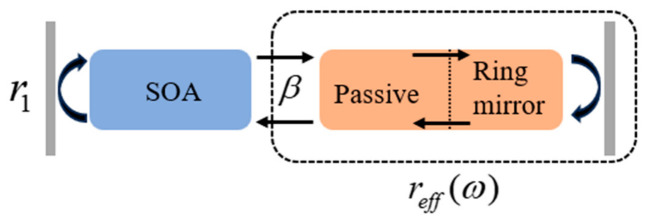
Equivalent model diagram of the ECL. The arrows indicate the optical propagation directions.

As illustrated in Figure 2, the proposed ECL can be described as a one-dimensional cavity composed of three sections. The left part corresponds to the active region, where the gain chip, together with the coating on its left facet, is replaced by an equivalent reflector with reflectivity $r_{1}$. The right-hand section, consisting of passive waveguides, a cascaded triple-ring resonator, and a Sagnac loop mirror, supplies wavelength-selective feedback and thereby establishes the passive external cavity. For analytical convenience, the region marked by the dashed box in Figure 2 is further reduced to a single effective mirror. The corresponding effective reflectivity, $r_{eff}(\omega )$, is determined by the product of the transfer functions of all passive components.

(3) $$r_{eff}(\omega )=\beta \cdot t_{passive}^{2}(\omega )\cdot r_{mirror}(\omega )$$

Here, β is introduced to represent the loss occurring at the interface between the active and passive sections, with a value of approximately −1 dB. The passive waveguide, whose overall length is $L_{p}$, can then be described by the following transfer function:

(4) $$t_{\mathrm{passive}}(\omega )=\exp(-\alpha_{p}L_{p}-j\beta_{p}L_{p})$$

where $\alpha_{p}$ and $\beta_{p}$ represent the electric-field attenuation in the waveguide and the corresponding effective propagation constant, respectively. If each ring is assumed to be symmetrically coupled, meaning that it has identical coupling ratios with the two bus waveguides, the mirror amplitude reflectivity is written as:

(5) $$t_{\mathrm{drop}}(\kappa_{m},\kappa_{m},R_{m},\alpha_{m})=\frac{-\kappa_{m}^{2}{\left(2\pi R_{m}\alpha_{m}\right)}^{1/2}e^{j2\pi R_{m}\beta_{p}/2}}{1-(1-\kappa_{m}^{2})2\pi R_{m}\alpha_{m}e^{j2\pi R_{m}\beta_{p}}}$$

(6) $$r_{mirror}(\omega )=-2j\sqrt{(1-\kappa_{c}^{2})\kappa_{c}^{2}}\cdot \prod_{m}t_{\mathrm{drop}}(\kappa_{m},\kappa_{m},R_{m},\alpha_{m})$$

Here, $t_{drop}$ denotes the field transmission coefficient from the input port to the drop port of the m-th ring (m=1,2,3) resonator, $\kappa_{m}$, $R_{m}$, and $\alpha_{m}$ represent the cross-coupling coefficient, the radius, and the amplitude attenuation constant of the m-th ring (m=1,2,3), respectively. The linewidth evaluation presented below is based on the theoretical treatment of external cavity semiconductor lasers reported by Patzak et al. [23]. and Kazarinov and Henry [24]. Owing to the frequency-dependent phase shift and reflectivity introduced by the extended passive cavity, the external cavity laser can achieve a considerably narrower linewidth than a standalone Fabry–Perot laser, with the linewidth reduction quantified by the linewidth-narrowing factor $F^{2}$, where F is the external cavity enhancement factor. On this basis, the Lorentzian linewidth of the laser is obtained analytically as:

(7) F=1+A+B

(8) A=1τ0Rejdlnreff(ω)dω=1τ0dφeffdω

(9) B=−αHτ0Imjdlnreff(ω)dω=αHτ0dln|reff(ω)|dω

(10) $$\Delta \nu_{0}=\frac{1}{4\pi }\frac{\nu_{g}^{2}h\nu n_{sp}\alpha_{tot}\alpha_{m}}{P_{0}[1+\frac{r_{1}}{\left|r_{eff}(\omega )\right|}\cdot \frac{1-|r_{eff}(\omega ){|}^{2}}{1-r_{1}^{2}}]}(1+\alpha_{H}^{2})$$

(11) $$\Delta \nu =\frac{\Delta \nu_{0}}{F^{2}}=\frac{\Delta \nu_{0}}{{\left(1+A+B\right)}^{2}}$$

where $\varphi_{eff}$ denotes the frequency-dependent phase of the effective reflectivity $r_{eff}(\omega )$, $\tau_{0}$ represents the photon round-trip time in the active gain section, $\alpha_{H}$ is the Henry linewidth enhancement factor, $\nu_{g}$ denotes the group velocity in the active gain section, h is Planck’s constant, ν denotes the laser frequency, and $n_{sp}$ denotes the spontaneous emission factor. In addition, $\alpha_{\mathrm{tot}}=\alpha_{i}+\alpha_{m}$ represents the overall loss, with $\alpha_{m}$ and $\alpha_{i}$ corresponding to the mirror loss and internal loss of the active section, respectively. $P_{0}$ is the output power of the laser, $\Delta \nu_{0}$ refers to the Schawlow–Townes linewidth of an isolated Fabry–Perot laser, and Δν denotes the linewidth of the ECL.

The linewidth-narrowing behavior is determined by the term F=1+A+B. In this expression, the factor A represents the resonance-induced extension of the optical phase path, whereas the factor B characterizes the strength of optical negative feedback and reflects the extent of phase perturbation within the external cavity. When the operating wavelength approaches the resonance of the cascaded three-ring reflector, the reflector provides the largest effective optical delay, making the contribution of A dominant. Meanwhile, lasing takes place near the center of the main reflection band, where the frequency slope of the reflection response is nearly zero, so the influence of B is greatly suppressed. This indicates that the observed linewidth reduction mainly originates from the longer effective external cavity rather than from feedback-related phase modulation. Moreover, high-Q rings exhibit narrower resonance linewidths and stronger wavelength selectivity, corresponding to a smaller full width at half maximum of the reflection spectrum. In the cascaded triple-ring structure, these properties help enhance the effective optical length and prolong the photon lifetime in the external cavity. Consequently, the laser linewidth can be substantially narrowed.

## 3. Device Design

### 3.1. Asymmetric Waveguide Bend

**Figure 3:**
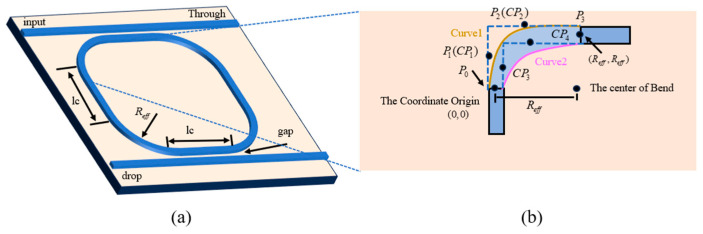
(**a**) Schematic diagram of a racetrack ring resonator. (**b**) Schematic diagram of the asymmetric waveguide bend.

Figure 3 illustrates the proposed asymmetric waveguide bend, which is defined by two independently designed boundary curves rather than a pair of symmetric edges. This configuration provides greater design freedom for bend optimization and helps achieve a smoother modal transition between the straight and bent waveguide sections. A flexible cubic Bezier curve with variable curvature is adopted as the optimization profile for the asymmetric waveguide bend, and the modified Bezier curve can be expressed as:

(12) $$P(t)={\left(1-t\right)}^{3}\cdot P_{0}+3t{\left(1-t\right)}^{2}\cdot P_{1}+3t^{2}(1-t)\cdot P_{2}+t^{3}\cdot P_{3},\;0\leq t\leq 1$$

where $P_{0}$ and $P_{3}$ are the two end points of the cubic Bezier curve, $P_{1}$ and $P_{2}$ are the control points, and P(t) represents the trajectory of the curve. Since the curve shape is determined by the positions of the control points, the above equation can be further rewritten in the two-dimensional parametric form as

(13) $$\left\{\begin{array}{c} x_{P}(t)=x_{P_{0}}\cdot {\left(1-t\right)}^{3}+3x_{P_{1}}\cdot t{\left(1-t\right)}^{2}+3x_{P_{2}}\cdot t^{2}(1-t)+x_{P_{3}}\cdot t^{3},\;0\leq t\leq 1\\ y_{P}(t)=y_{P_{0}}\cdot {\left(1-t\right)}^{3}+3y_{P_{1}}\cdot t{\left(1-t\right)}^{2}+3y_{P_{2}}\cdot t^{2}(1-t)+y_{P_{3}}\cdot t^{3},\;0\leq t\leq 1 \end{array}\right.$$

where $\left(x_{P_{i}},y_{P_{i}}\right)$ denote the two-dimensional coordinates of point $P_{i}$ (i=0,1,2,3). Therefore, the shape of the Bezier curve is mainly determined by the positions of the control points. For the proposed asymmetric waveguide bend, the two boundary curves are independently defined by two sets of control points: Curve 1 contains $CP_{1}(c_{1x},c_{1y})$ and $CP_{2}(c_{2x},c_{2y})$, while Curve 2 contains $CP_{3}(c_{3x},c_{3y})$ and $CP_{4}(c_{4x},c_{4y})$. To ensure a smooth connection between the bent and straight waveguides while maintaining the same device footprint, the control points of a bend with an effective radius $R_{eff}$ and a waveguide width W are subject to the following geometric constraints:

(14) $$\left\{\begin{array}{c} c_{1x}=0\\ c_{3x}=0\\ c_{2y}=R_{eff}+\frac{W}{2}\\ c_{4y}=R_{eff}-\frac{W}{2}\\ c_{1y}\in [0,R_{eff}+\frac{W}{2}]\\ c_{3y}\in [0,R_{eff}-\frac{W}{2}]\\ c_{2x}\in [0,R_{eff}+\frac{W}{2}]\\ c_{4x}\in [0,R_{eff}-\frac{W}{2}] \end{array}\right.$$

**Figure 4:**
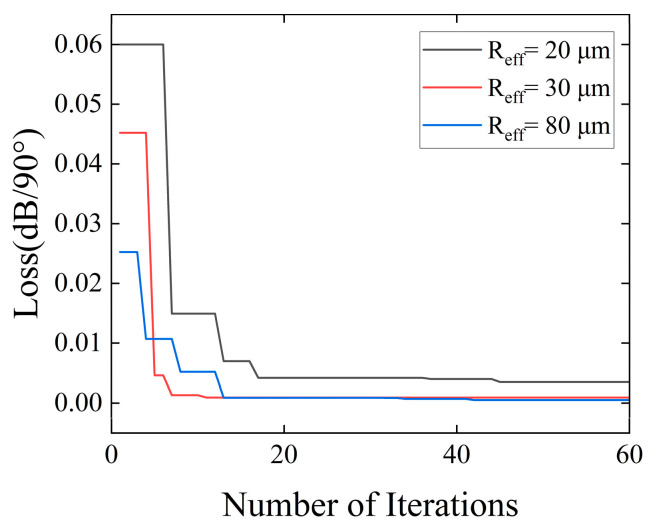
Loss evolution during PSO of the modified Si_3_N_4_ Bezier bend with effective radii of 20, 30, and 80 μm.

Under these constraints, the key parameters to be determined by the PSO algorithm are $c_{1y}$, $c_{2x}$, $c_{3y}$ and $c_{4x}$. The values of these parameters are summarized in Table 1, and the corresponding optimization and sweep results are shown in Figure 4.

The simulation results are summarized in Table 2, which shows that the asymmetric waveguide bend consistently outperforms the circular bend in terms of bend loss for all considered $R_{eff}$ values.

### 3.2. Laser Structure

**Figure 5:**
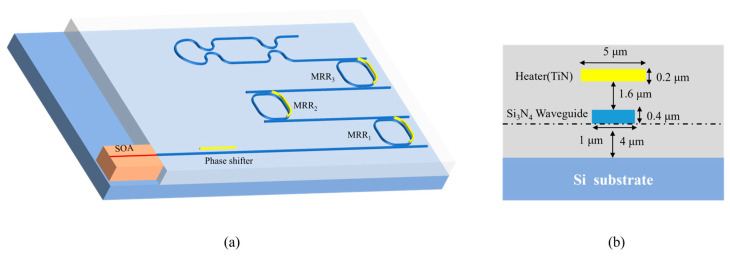
Schematic of the cascaded triple-ring ECL. (**a**) Device architecture of the cascaded triple-ring ECL. (**b**) Waveguide cross-section with a TiN heater.

Figure 5a presents the architecture of the proposed hybrid ECL, where a semiconductor optical amplifier (SOA) is coupled to a passive Si_3_N_4_ feedback circuit through precise chip alignment. The passive section contains two key elements, namely a cascaded three-ring filtering unit and a Sagnac loop reflector, whereas optical amplification is provided by the SOA chip. The Sagnac loop reflector is located after the cascaded triple-ring filter and is formed by a directional-coupling region and a looped Si_3_N_4_ waveguide. The wavelength-selected optical field entering the loop is divided into two counter-propagating components, which recombine in the coupling region after propagating along the loop. The interference between these two components determines the reflected and transmitted fields. A cross-sectional view of the Si_3_N_4_ resonator region is given in Figure 5b. For thermal control of the resonance, TiN heaters are fabricated above the ring waveguides. Each heater is 0.2 μm thick, 5 μm wide, and positioned 1.6 μm above the resonator. In the gain chip, the active region is a 400 μm multiple-quantum-well structure, and light confinement is achieved with an InP ridge waveguide. The SOA facet away from the external cavity has a reflectivity of 0.3. By contrast, the facet adjacent to the passive chip employs a tilted-waveguide design together with an anti-reflection coating, which suppresses the reflectivity to 1 × 10^−4^. In addition, the Sagnac loop reflector is designed with a reflectivity of 0.9. When current is injected into the SOA, amplified light is launched into the passive Si_3_N_4_ chip, spectrally selected by the cascaded ring filter, and then sent back by the Sagnac reflector. A fraction of this returned field is coupled back into the SOA to sustain the external cavity, while the remaining fraction serves as the output of the laser.

## 4. Numerical Analysis

### 4.1. Asymmetric Bezier Racetrack Resonators

**Figure 6:**
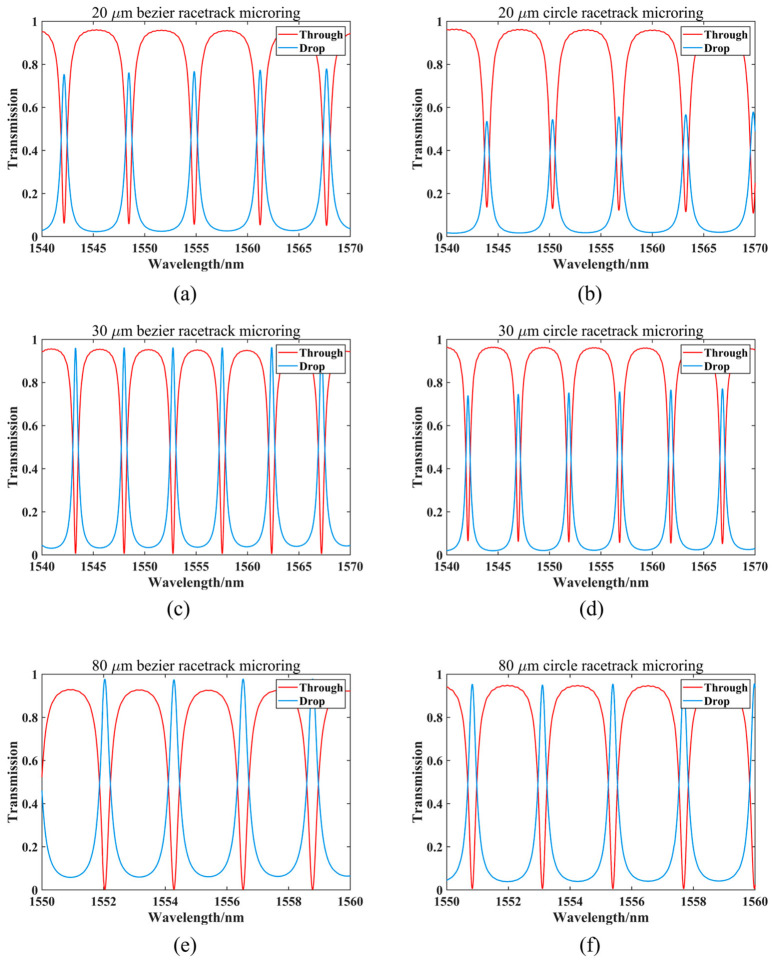
Comparison of the simulated transmission spectra of asymmetric Bezier and circular racetrack rings with effective bend radii of 20 μm, 30 μm, and 80 μm: (**a**) 20 μm asymmetric Bezier racetrack ring; (**b**) 20 μm circular racetrack ring; (**c**) 30 μm asymmetric Bezier racetrack ring; (**d**) 30 μm circular racetrack ring; (**e**) 80 μm asymmetric Bezier racetrack ring; and (**f**) 80 μm circular racetrack ring.

To evaluate the influence of the proposed asymmetric Bezier bend on the resonator performance, the transmission spectra of the designed resonant rings are simulated and compared with those of a reference structure. For a fair comparison, the two structures are designed with the same straight-waveguide length and coupling gap, where the straight-waveguide length $L_{c}$ is set to 20 μm and the coupling gap is fixed at 0.7 μm, while only the bend profile is modified. The bend radii considered in the simulation are 20 μm, 30 μm and 80 μm, respectively. Figure 6 shows the simulated spectra of the reference resonant ring and the asymmetric Bezier resonant ring. From these spectra, the resonance wavelength, the 3 dB bandwidth, and the minimum transmission at resonance can be extracted to evaluate the intrinsic quality factor of the resonator. The intrinsic quality factor $Q_{i}$ is expressed as follows:

(15) $$Q_{i}=\frac{\lambda_{res}}{\Delta \lambda_{3\mathrm{dB}\_drop}\sqrt{\gamma_{through}}}$$

where $\lambda_{res}$ is the resonant wavelength of the ring resonator, $\Delta \lambda_{3\mathrm{dB}\_drop}$ is the 3 dB bandwidth of the resonance response, and $\gamma_{through}$ denotes the minimum normalized transmission at resonance. The extracted parameters, including the resonance wavelength, the 3 dB bandwidth of the drop port, and the minimum transmission at the through port, together with the calculated intrinsic quality factors of the asymmetric Bezier resonant ring and the reference circular resonant ring, are summarized in Table 3.

Compared with the circular resonator, the racetrack ring resonator with the asymmetric Bezier bend exhibits superior performance for all investigated bend radii. Specifically, the minimum through-port transmission $\gamma_{through}$ is reduced, indicating a lower round-trip loss in the resonator. At the same time, the intrinsic quality factor $Q_{i}$ is significantly improved. For bend radii of 20 μm, 30 μm, and 80 μm, the $Q_{i}$ values of the asymmetric bend resonator are 9251, 38,122, and 137,530, respectively. In contrast, the corresponding $Q_{i}$ values of the circular resonator are 5238, 11,012, and 60,516, respectively. These results indicate that the proposed asymmetric bend can effectively suppress bend-induced loss without increasing the device footprint. As the bend radius increases, the $Q_{i}$ values of both structures become higher due to the reduction in bending loss. However, the asymmetric bend design still consistently outperforms the conventional circular design. The proposed asymmetric Bezier bend provides an effective approach for realizing compact racetrack resonators with high quality factors.

### 4.2. Spectrum of the Cascaded Triple-Ring Filter

**Figure 7:**
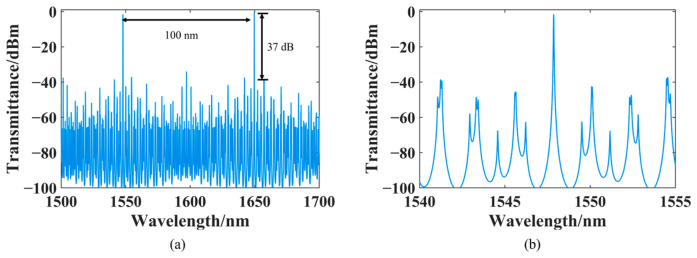
(**a**) Reflection spectra of the three-ring external cavity. The spectrum has a tuning range of 100 nm and an SMSR of 37 dB. (**b**) Enlarged image of the resonance peak.

The effective radii of the three rings are 80 μm, 82 μm, and 115 μm for $R_{1}$, $R_{2}$ and $R_{3}$, respectively. Based on these parameters, the spectral response of the cascaded triple-ring filter is shown in Figure 7. The filter provides an effective tuning range of 100 nm with an SMSR of 37 dB. As shown in Figure 7b, the main resonance peak of the proposed asymmetric Bezier triple-ring filter exhibits a normalized peak intensity of about −2 dB. For comparison, the corresponding peak intensity of a conventional circular-ring design with the same ring circumferences is estimated to decrease to about −3 dB.

### 4.3. Theoretical Linewidth Calculation for Lasers

**Figure 8:**
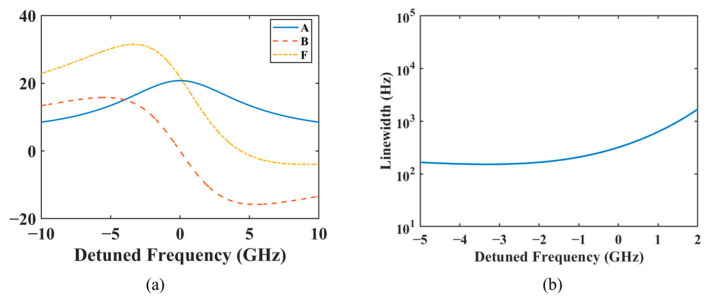
(**a**) Calculated values of coefficients A, B and F; and (**b**) Estimated Lorentzian linewidth at 20 mW output power.

Using the framework developed in Section 2 together with the parameter set given in Table 4, we determine the frequency-dependent behavior of the three coefficients around the 1547 nm lasing mode. Here, detuning is defined as the frequency offset between the longitudinal mode of the gain cavity and the resonance frequency of the external cavity filter. Their variation with detuning is presented in Figure 8a. The maximum of the B factor occurs at the rising edge of the resonance frequency, whereas the A factor exhibits a symmetric distribution with its peak located at the resonance frequency. Therefore, the F factor reaches its maximum at a frequency slightly deviated from resonance. Using Equation (11), the corresponding Lorentzian linewidth is calculated and shown in Figure 8b. The minimum linewidth is approximately 234 Hz near the lasing mode, indicating a sub-kHz linewidth predicted by the analytical model.

**Figure 9:**
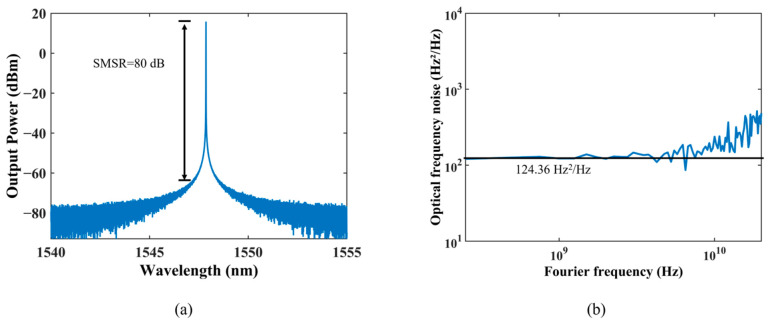
Characterization of the laser in terms of the optical spectrum and frequency noise. (**a**) Emission spectrum of the laser output, exhibiting an SMSR of 80 dB; and (**b**) simulated frequency noise spectrum at 1547 nm.

To further evaluate the proposed laser, the SOA was simulated using the traveling-wave laser model (TWLM), and the overall ECL was analyzed in the transient sample mode. The simulation results are shown in Figure 9. At a pump current of 100 mA, the laser is centered at 1547 nm and delivers an output power of 34 mW, while the simulated optical spectrum indicates an SMSR of more than 80 dB. Moreover, the simulated frequency noise spectrum exhibits a white-noise floor of 124.36 Hz^2^/Hz, from which a Lorentzian linewidth of 390 Hz can be derived.

**Figure 10:**
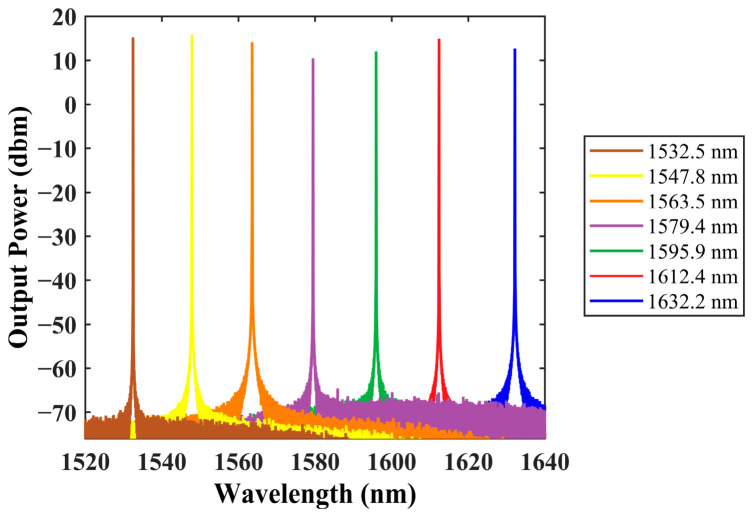
Simulated optical spectra at different operation wavelengths.

The wavelength tuning behavior of the proposed laser was then examined by thermally adjusting the MRR heaters, and the tuning map was generated while the gain current was held constant at 100 mA during the entire simulation process. Figure 10 presents the optical spectra corresponding to several lasing wavelengths. It is found that the laser can cover a tuning interval of 100 nm, while preserving an SMSR above 80 dB and a sub-kHz linewidth across the full range. Table 5 summarizes the simulated linewidth, SMSR, and output power at several representative wavelengths.

## 5. Discussion

Table 6 provides an overview of the main performance parameters reported for earlier ECLs together with those obtained in this work. Dual-ring ECLs [12,13,14,25] show different advantages in specific aspects, yet it is still difficult for them to achieve balanced performance in linewidth, tuning range, SMSR, and output power at the same time. A typical example is Ref. [25], which delivers a wavelength tuning span of 172 nm and an output power of 26.7 mW. Nevertheless, its linewidth is still 4 kHz and the SMSR reaches only 40 dB. Triple-ring ECLs [26,27,28], in contrast, introduce an additional resonant cavity and therefore offer greater flexibility in spectral engineering. Their main advantage is that the third ring can further suppress side modes while preserving a wide tuning range, which helps achieve more balanced overall performance than typical dual-ring structures. Nevertheless, previously reported triple-ring schemes still show clear weaknesses. In particular, their output power is generally limited, and in some cases the linewidth or tuning range is still not ideal. For example, Ref. [26] achieves a tuning range of 96 nm, but the SMSR is only 42 dB and the output power is just 0.98 mW.

In this context, the advantage of the present work does not arise solely from the triple-ring cavity itself. Recent simulation-based triple-ring ECL designs have improved laser performance mainly from two aspects. On the one hand, Vernier ring radius optimization has been employed to improve the spectral alignment among multiple rings, thereby enhancing the tuning range and SMSR [29]. However, this approach mainly optimizes the resonance spacing and side-mode distribution of the cascaded rings, while the bend-induced loss and quality factor of each individual resonator are not directly addressed. On the other hand, Euler-type resonant rings have been introduced to smooth the bend transition and reduce bending loss in narrow-linewidth ECLs [20]. Nevertheless, Euler bends are generally designed by optimizing the waveguide centerline, and the two boundaries of the curved waveguide are still not independently controlled. Therefore, residual mode mismatch between the straight and curved waveguide sections and the footprint–loss trade-off may still limit further improvement of the resonator quality factor.

To overcome these limitations, this work further introduces an asymmetric Bezier bend into the cascaded triple-ring ECL. The asymmetric Bezier bend provides greater geometric freedom than conventional circular or Euler-type bends by independently optimizing the two boundaries of the curved waveguide. As a result, the intrinsic quality factor of the racetrack resonator is improved without significantly increasing the device footprint. Together with the cascaded triple-ring structure, which enhances wavelength selectivity, suppresses side modes, and increases the effective optical length of the external cavity, the improved racetrack resonator quality factor enables a more balanced combination of linewidth, tuning range, SMSR, and output power. The proposed asymmetric Bezier cascaded triple-ring ECL achieves a linewidth of 390 Hz, a tuning range of 100 nm, an SMSR exceeding 80 dB, and an output power of 34 mW.

Several aspects still merit further investigation in future work. First, the control-point parameters of the asymmetric Bezier bend can be further optimized to achieve a higher intrinsic quality factor over a wider range of bend radii. Second, the optical mode matching at the SOA–S_i3_N_4_ interface can be further improved through optimized taper design and transition structures to reduce insertion loss and back reflection. In addition, fabrication tolerance, thermal crosstalk among the ring heaters, and experimental validation should be further investigated to evaluate the practical feasibility of the proposed design.

## 6. Conclusions

We propose an AI-assisted ECL design based on an asymmetric bend racetrack ring resonator to achieve wide wavelength tunability, narrow linewidth, and a compact device footprint. By employing PSO to optimize the asymmetric Bezier bend, the intrinsic quality factor of the resonator is effectively improved under the same footprint constraints. The laser is predicted to achieve a Lorentzian linewidth of 390 Hz, an output power of 34 mW, and an SMSR exceeding 80 dB at 1547 nm. In addition, a wavelength tuning range of 100 nm is obtained, while the linewidth remains within the kilohertz range over the full tuning range. These results indicate that the proposed design is promising for compact, high-performance tunable laser sources.

With the continuous development of integrated photonics, high-performance laser sources with narrow linewidth and wide tunability are becoming increasingly important for a variety of applications. The asymmetric bend racetrack ring resonator presented in this work provides an effective approach for improving the performance of ECLs. It is expected that, with further advances in device optimization and fabrication technology, this design can offer useful guidance for the development of narrow-linewidth, widely tunable integrated laser sources.

## Figures and Tables

**Table 1:** Parameters of the silicon nitride asymmetric waveguide bend.

Parameters	$R_{eff}$		
	20	30	80
$c_{1y}$ (μm)	14.4	16.8	14.4
$c_{2x}$ (μm)	4.5	1.2	2.6
$c_{3y}$ (μm)	14.8	10.8	7.4
$c_{4x}$ (μm)	5.9	0	1.2

**Table 2:** Comparison of simulation results for waveguide bend loss.

Waveguide Bend Type	$R_{eff}$ (μm)		
	20	30	80
Asymmetric Waveguide Bend (dB/90°)	0.0015	0.0009	0.0004
Circular bend loss (dB/90°)	0.019	0.006	0.001

**Table 3:** Comparison of asymmetric Bezier and circular racetrack ring resonators.

	Type of Racetrack Ring Resonator					
	Asymmetric Bend			Circular		
$R_{eff}$ (μm)	20	30	80	20	30	80
$\lambda_{res}$ (nm)	1567.68	1567.22	1567.85	1563.61	1566.82	1569.24
$\Delta \lambda_{3\mathrm{dB}\_drop}$ (nm)	0.75	0.65	0.38	0.9	0.63	0.41
$\gamma_{through}$	0.051	0.004	0.0009	0.11	0.051	0.004
$Q_{i}$	9251	38,122	137,530	5238	11,012	60,516

**Table 4:** Structural parameters of the rings.

Parameter	Description	Value
κ^2^	Coupling coefficient	0.09
lc	Length of straight waveguide	20 μm
*R*_1_	Ring1 effective radius	80 μm
*R*_2_	Ring2 effective radius	82 μm
*R*_3_	Ring3 effective radius	115 μm
$\alpha_{H}$	Linewidth enhancement factor	2
$n_{sp}$	Spontaneous emission coefficient	2
$\alpha_{i}$	Internal loss of the active section	43 dB/cm

**Table 5:** Simulated performance of the ECL at different wavelengths.

Wavelength (nm)	Linewidth (Hz)	SMSR (dB)	Output Power (dBm)
1532.5	511	79.71	15.24
1547.8	390	80.31	15.31
1563.5	602	78.51	14.01
1579.4	930	75.95	11.27
1595.9	744	76.48	12.25
1612.4	453	79.41	14.84
1632.2	442	77.21	13.21

**Table 6:** Performance Comparison.

References	Type	Result Type	Linewidth (Hz)	Tuning Range (nm)	SMSR (dB)	Power (mW)
[12]	dual-ring	Experimental	80,000	55	50	220
[13]	dual-ring	Experimental	6.03	40	64.3	4.8
[14]	dual-ring	Experimental	23,800	90	50	1.4
[25]	dual-ring	Experimental	4000	172	40	26.7
[26]	triple-ring	Experimental	-	96	42	0.98
[27]	triple-ring	Experimental	17,500	30	55	-
[28]	triple-ring	Experimental	220	110	55	3
[20]	triple-ring	Simulation	300	100	30	-
[29]	triple-ring	Simulation	644	106	80	14.1
This work	triple-ring	Simulation	390	100	80	34

## Data Availability

The raw and processed data required to reproduce the findings of this study are not publicly available at this time, as they also form part of an ongoing extended study. Interested researchers may request access to the datasets from the corresponding author, Wen Zhou (email: zwen@fudan.edu.cn), subject to reasonable conditions and approval.
